# 胸腔镜辅助肺癌切除术后生活质量的研究

**DOI:** 10.3779/j.issn.1009-3419.2014.03.05

**Published:** 2014-03-20

**Authors:** 剑 曾, 金石 刘

**Affiliations:** 310004 杭州，浙江省肿瘤医院胸部肿瘤外科 Department of Thoracic Tumor Surgery, Zhejiang Cancer Hospital, Hangzhou 310004, China

**Keywords:** 肺肿瘤, 生命质量, 胸腔镜, Lung neoplasms, Quality of life, Thoracoscopy

## Abstract

**背景与目的:**

传统的肺癌根治手术创伤大，胸腔镜下手术能减少手术创伤，有望改善肺癌患者术后生命质量（quality of life, QOL）。本研究旨在探讨胸腔镜下肺癌根治术对患者QOL的影响。

**方法:**

使用由欧洲癌症研究与治疗组织（European Organization for Research and Treatment, EORTC）开发的质量核心问卷量表（Quality of Life-Core 30 Questionnaire, QLQ-C30）和针对肺癌患者的补充量表（Quality of Life-Lung Cancer 13 Questionnaire, QLQ-LC13）对60例分别接受胸腔镜和常规开胸肺癌根治术的非小细胞肺癌（non-small cell lung cancer, NSCLC）患者从术前3天至术后24周内的QOL进行评价。

**结果:**

60例患者一共收回问卷215份，其余25份失访。所有患者的QOL在术后3天时均出现明显下降，常规开胸组和胸腔镜组术前、后3天的总体QOL平均得分分别为：87.8±10.3 *vs* 38.3±13.1（*P* < 0.001）和82.7±9.6 *vs* 56.3±14.8（*P* < 0.001）。随后，所有患者的QOL开始回升。常规开胸组患者的QOL下降更为明显，术后3天的总体QOL得分明显低于胸腔镜组（*P*=0.012, 9），术后24周时仍然不能恢复至术前水平（*P*=0.0124）。胸腔镜组术后QOL下降相对较小，术后24周时大部分指标恢复至术前水平。

**结论:**

胸腔镜下肺癌根治术创伤小，恢复快，术后QOL高于传统开胸手术，是一种较为可取的肺癌术式。

## Abstract (en)

**Background and objective:**

Conventional radical surgeries for lung cancer incur a large amount of trauma, thoracoscopic surgeries can reduce trauma, and hopefully improve patients' postoperative quality of life (QOL). The aim of this study is to evaluate the impact of thoracoscopic radical surgeries on quality of life of patients with non-small cell lung cancer (NSCLC).

**Methods:**

Use Quality of Life-Core 30 Questionnaire (QLQ-C30) which was exploited by European Organization for Research and Treatment (EORTC) and Quality of Life-Lung Cancer 13 Questionnaire (QLQ-LC13) which is the supplementary questionnaire according to the lung cancer patients to evaluate QOL of 60 NSCLC patients in thoracoscopic surgery group and conventional surgery group from 3 days before operation to 24 weeks after operation.

**Results:**

A total of 215 questionnaires were collected from 60 patients; 25 postoperative questionnaires were not completed because patients could not be contacted for follow-up visits. QOL declined markedly in all patients at 3 days postoperatively; preoperative and 3-day postoperative global QOL scores in the conventional surgery and thoracoscopic surgery group were 87.8±10.3 *vs* 38.3±13.1 (*P* < 0.001) and 82.7±9.6 *vs* 56.3±14.8 (*P* < 0.001), respectively. Thereafter, QOL recovered gradually in all patients. Patients who underwent open surgery showed the most pronounced decline in QOL; global scores were lower in this group than in the thoracoscopic surgery group (*P*=0.012, 9) at 3 days postoperatively and was not restored to the preoperative level at 24 weeks postoperatively (*P*=0.012, 4). QOL declined less in patients undergoing thoracoscopic surgery, and most indices had recovered to preoperative levels at 24 weeks postoperatively.

**Conclusion:**

With the advantages of small trauma, faster recovery and higher postoperative QOL, thoracoscopic surgery is a preferable lung cancer surgery.

传统的肺癌根治手术创伤大，术后生命质量（quality of life, QOL）差。近年来，以胸腔镜手术为代表的肺癌微创外科的发展，给提高肺癌患者术后QOL带来了希望。我们研究了本院近一年来接受胸腔下肺癌根治术的非小细胞肺癌（non-small cell lung cancer, NSCLC）患者术前以及术后短期内的QOL情况，并与传统开胸手术作对比，汇报如下。

## 1 资料与方法

### 1.1 资料

浙江省肿瘤医院2012年1月-2012年12月接受手术治疗的NSCLC患者共60例，男性46例，女性14例，年龄46岁-81岁。术前均未接受过放疗或化疗，肿瘤未侵及胸壁、大血管或叶支气管根部，无需行胸壁切除、多叶肺切除或支气管袖式成型，无伴发影响QOL的内科疾病。按入院先后顺序随机分为二个组：胸腔镜组和常规开胸组，每组均为30例（表 1）。

**1:** 60例接受手术的非小细胞肺癌患者临床资料 Clinical data of 60 patients undergoing surgeries for non-small cell lung cancer (NSCLC)

Clinical data	Surgical approach	
	Thoracoscopic	Conventional open
Patients (*n*)	30	30
Mean age (year）	53.2±13.4	62.3±9.1
Gender (*n*)		
Male	19	5
Female	21	25
Tumor diameter (cm)	1.7±1.4	1.5±0.8
Tumor location (*n*)		
Right upper lobe	11	9
Right middle lobe	2	1
Right lower lobe	7	10
Left upper lobe	5	6
Left lower lobe	5	4

### 1.2 方法

#### 1.2.1 手术方式

所有患者均行肿瘤所在肺叶切除加系统性淋巴结清扫。

##### 1.2.1.1 胸腔镜组

患者取侧卧位，取腋中线第7肋间为胸腔镜观察口，先进腔镜头，在腔镜观察下调节操作孔定位。取腋前线第4肋间为主操作孔，腋后线第7肋间为副操作孔。探查后，首先游离肺叶间裂，显露肺动脉并分别缝扎处理肺叶动脉各分支。游离肺叶静脉，使用腔镜下血管切割闭合器处理。最后游离支气管，用腔镜下支气管切割闭合器处理。若病变位于右肺，则清扫第2、4、7、8、9、10、11组淋巴结；若病变位于左肺，则清扫第5、6、7、8、9、10、11组淋巴结。

##### 1.2.1.2 常规开胸组

患者取侧卧位，取胸后外侧切口，长约10 cm。断离部分背阔肌，沿肌肉走行撕开部分前锯肌，以第4肋间进胸。按病变所在肺叶分别缝扎处理肺动、静脉。支气管闭合器处理支气管。淋巴结清扫范围同胸腔镜组。

#### 1.2.2 化疗

病理分期为Ⅰb及以上者，术后予4个疗程含铂方案化疗。

#### 1.2.3 QOL评测

使用由EORTC开发的质量核心问卷量表质量（Quality of Life-Core 30 Questionnaire, QLQ-C30）以及其针对肺癌患者开发的补充量表（Quality of Life-Lung Cancer 13 Questionnaire, QLQ-LC13），对患者QOL进行评价。

调查时间分别为术前3天及术后3天、12周、24周。前二次为患者住院期内面访，后二次为电话询问的方式。问卷由患者本人作答，有阅读障碍的患者则由医生或其家属向其讲明问卷内容，按患者回答代填。

### 1.3 统计学处理

量表各项目按其计分方法经线性变换成1-100的得分，算出各组的平均值和标准差。数据采用SPSS 13.0软件处理，组间差异采用*χ*^2^检验或独立样本*t*检验。*P* < 0.05为差异有统计学意义。

## 2 结果

### 2.1 两组患者术前临床资料比较

包括年龄、性别、肿瘤大小、部位均无明显差别（表 1）。

### 2.2 两组患者术中情况比较

用时、出血、淋巴结清扫个数、术后病理类型和TMN分期均未见明显差别。胸腔镜组术后发生肺部感染2例，声音嘶哑1例。常规开胸组术后发生肺部感染3例，术后出血1例。两组患者术后并发症发生率无统计学差异，经治疗后均全愈（表 2）。在术后24周的随访中，患者均存活，亦未见肿瘤复发或转移。

**2:** 60例患者术中以及术后指标 Intra- and post-operative variables in 60 patients

	Surgical approach	
	Thoracoscopic	Conventional open
Operation time (min)	123±23	109±19
Blood loss (mL)	118±57	126±89
Postoperative complications (*n*)		
Postoperative bleeding	0	1
Lung infection	2	3
Hoarseness	1	0
Pathological types (*n*)		
Adenocarcinoma	19	22
Squamous cell carcinoma	11	8
Lymph node dissection number	38±15	42±17
Stage (*n*)		
Ⅰ	17	15
Ⅱ	11	12
Ⅲ	2	3
Ⅳ	0	0

### 2.3 问卷结果比较

60例患者一共收回问卷215份，均符合答题要求，其余25份术后失访。两组患者在术前3天QOL各指标无明显差别。术后3天较术前相比，QOL均明显下降。常规开胸组和胸腔镜组术前、后3天的总体生命质量平均得分分别为：87.8±10.3 *vs* 38.3±13.1（*P* < 0.001）和82.7±9.6 *vs* 56.3±14.8（*P* < 0.001）（图 1A）。大部分功能性指标得分降低，其中躯体功能、社会功能、角色功能、情绪功能指标得分下降最为明显（图 1B-E）；大部分症状性指标，如虚弱、疼痛、咳嗽和气促指标得分上升（图 1F-G、图 2A-B）。常规开胸组QOL下降较明显，相比胸腔镜组在躯体功能、情绪功能、疼痛等指标等分均有统计学差异（*P* < 0.001）（图 1B、E、G）。

**1:**
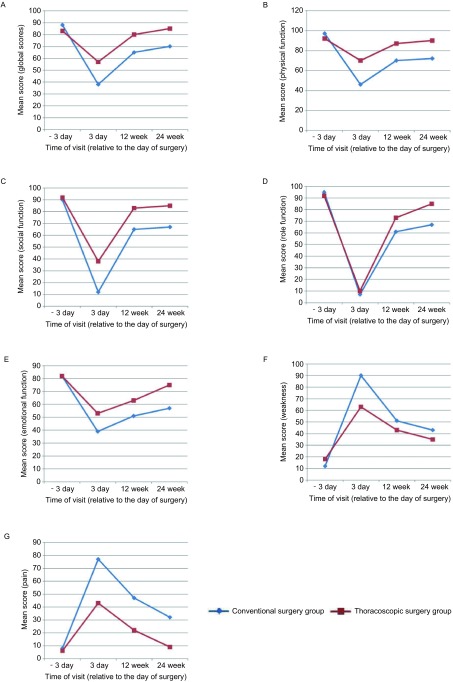
两组患者分别在术前3天、术后3天、12周和24周QLQ-C30量表得分情况。A：总体QOL；B：躯体功能；C：社会功能；D：角色功能；E：情绪功能；F：虚弱；G：疼痛。 QOL, as determined by quality of life-core 30 questionnaire scores at 3 days before and 3 days, 12, 24 weeks after surgery, in patients with NSCLC undergoing thoracoscopic and conventional surgery. A: Global QOL; B: Physical function; C: Social function; D: Role function; E: Emotional function; F: Weakness; G: Pain; QOL: Quality of life.

随着术后时间的推移，两组患者QOL均逐渐提高。在术后12周，两组患者各项指标已逐渐恢复。胸腔镜组患者在术后12周和24周时，总体QOL得分均高于传统开胸组（*P*=0.0269; *P*=0.0301），后者在术后24周时仍然不能恢复至术前水平（*P*=0.0124）（图 1A）。术后24周，胸腔镜组大部分功能性指标已回恢复致术前水平，但常规开胸组较术前仍有较大差距。

另外，两组中均有约1/3的患者出现术后慢性干咳症状，得分无统计学差异（图 2A）。

**2:**
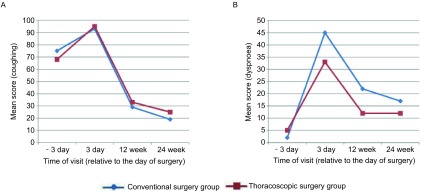
两组患者分别在术前3天、术后3天、12周和24周时使用QLQ-LC13量表测得的肺癌相关性症状得分情况。A：咳嗽；B：气促。 Severity of symptoms associated with lung cancer, as determined by quality of life-lung cancer 13 questionnaire (QLQ-LC13) scores at 3 days before and 3 days, 12, 24 weeks after surgery, in patients with NSCLC undergoing thoracoscopic and conventional surgery. A: Coughing; B: Dyspnoea.

## 3 讨论

QOL由世界卫生组织（World Health Organization, WHO）定义为：不同文化和价值体系中的个体对他们的目标、期望、标准以及所关心的事情有关的生存状况体验。质量核心问卷量表QLQ-C30已被应用于多种癌症患者QOL的研究，它是一种适用于各种癌症患者的标准问卷^[1]^。QLQ-LC13针对性的应用于肺癌患者，是核心量表QLQ-C30的补充量表。QLQ-C30功能性指标及总体QOL指标分值越高提示功能越好、QOL越高；QLQ-C30症状性指标及QLQ-LC13肺癌症状指标得高越高则症状越明显，QOL越低^[2]^。Park等^[3]^使用QLQ-C30和QLQ-LC13量表前瞻性分析了接受辅助化疗的老年NSCLC患者QOL情况，认为此量表具有较好的可靠性。Schulte等^[4]^使用QLQ-C30和QLQ-LC13评估了老年NSCLC患者术后QOL情况，发现相对年青患者，老年患者术后QOL恢复慢，多数难以恢复至术前水平。国内学者Cheng等^[5]^使用中文版QLQ-C30和QLQ-LC13对106例肺癌患者的QOL进行评估，发现年龄、性别、疾病分期、治疗方法以及教育和收入水平都是影响QOL的因素。目前的国内外研究表明，QLQ-C30和QLQ-LC13联合使用能较客观的反映肺癌患者的QOL。

传统的肺癌开放性手术切口较大，同时需切断肋骨，使用肋骨撑开器，离断部分背阔肌并损伤前锯肌。所以，在治疗的同时也给患者带来很大的创伤，不利于术后恢复和术后QOL。本研究中，常规开胸组在术后患者QOL明显下降，术后24周仍有多个指标恢复不理想，如总体QOL、躯体功能指标、社会功能指标、情绪功能指标和疼痛等。

近10年来，随着技术和设备的发展，肿瘤治疗理念的更新，特别是新一代腔镜成像系统的问世，为在胸腔镜下进行肺叶切除和淋巴结清扫提供了有利条件。目前，胸腔镜应用于肺癌的外科治疗已经广泛开展，手术经验技巧日趋丰富和完善，且取得良好的临床疗效^[6-9]^。我院自2010年初开展胸腔镜下肺癌根治术以来，已完成手术200余例，效果令人满意。在本研究中，我们发现胸腔镜组患者术后QOL下降少，恢复快。因切口小，避免大面积断离肌肉，胸腔镜下术后24周，大部分患者躯体功能指标恢复至术前水平。同时，胸腔镜组患者角色功能、情绪功能、社会功能恢复亦比常规开胸组好。在随访中我们了解，许多患者因术后的手术疤痕影响到社会功能和角色功能的得分，因为胸腔镜手术的疤痕小，有利患者自信心的恢复，这可能是胸腔镜组的这两项功能指标得分高于常规开胸组的重要原因。症状指标中，胸腔镜组患者在术后疼痛、虚弱、气促等明显较于常规开胸组轻。胸外科术后慢性胸痛一直是影响患者QOL的一个重要因素，报道^[10]^显示约25%-60%胸外科术后患者出现慢性胸痛，肋间神经的损伤可能是其主要因素。本研究中，胸腔镜组患者因手术切口小，不使用肋骨撑开器，减少了肋间神经损伤的机率，术后慢性胸痛的发生率大大降低。

我们的研究发现，肺癌术后干咳是严重影响患者QOL的一个因素，不论是胸腔镜组还是常规开胸组，均有相当一部分患者术后发生慢性干咳，当空气干燥、气温较低时更易诱发。肺癌术后慢性咳嗽原因可能在于纵隔淋巴结清扫^[11]^。还有学者^[12]^认为，术中气管下段以及支气管的刺激是干咳的主要原因。我们发现，术后慢性干咳予常规止咳药往往效果不佳，嘱患者注意保暖和空气湿化对部分患者有效，但目前仍是一个尚待解决的问题。

胸腔镜技术应用于NSCLC的外科治疗目前已得到公认并被美国国立综合癌症网络（National Comprehensive Cancer Network, NCCN）指南推荐为可选术式，但多数应用于临床分期较早，肿瘤位于外周的病例。随着手术经验的积累和手术技巧的日趋成熟，胸腔镜下肺癌根治术后适用范围也逐浙扩大。本研究中，胸腔镜组Ⅱ期以上患者约占40%，其中2例为Ⅲa期。虽然此类患者胸腔镜下术后远期疗效尚待观察，但在本研究中，其手术的切除范围和淋巴结清扫个数与常规开胸性手术并无差异，与文献^[13, 14]^报道相仿。而且我们认为，胸腔镜手术创伤小，术后恢复快，QOL高，有利于术后辅助治疗的耐受性和依从性，从而有望提高综合治疗效果。已有报道^[15-17]^显示，肺癌术后QOL和远期生存率呈正比。结合本研究结果，我们认为，对于可切除的NSCLC，若设备和技术条件允许，应尽量选择胸腔镜下手术，以提高患者术后QOL。

本研究中部分患者术后接受辅助化疗，可能给短期QOL带来负面影响，此为本研究的一个干扰因素。同时，本研究随访工作仅延续至术后半年，病例数较少，远期效果及生存率有待加大样本量进一步观察。
